# The Timing and Effects of Low-Dose Ethanol Treatment on Acetaminophen-Induced Liver Injury

**DOI:** 10.3390/life11101094

**Published:** 2021-10-15

**Authors:** Fu-Chao Liu, Huang-Ping Yu, Chia-Chih Liao, An-Hsun Chou, Hung-Chen Lee

**Affiliations:** 1 Department of Anesthesiology, Chang Gung Memorial Hospital, Taoyuan 333, Taiwan; ana5189@cgmh.org.tw (F.-C.L.); yuhp2001@cgmh.org.tw (H.-P.Y.); m7147@cgmh.org.tw (C.-C.L.); f5455@cgmh.org.tw (A.-H.C.); 2 College of Medicine, Chang Gung University, Taoyuan 333, Taiwan

**Keywords:** acetaminophen, ethanol, alcohol, liver injury, inflammation, neutrophil, cytochrome P450, CYP2E1

## Abstract

Acetaminophen (APAP) overdose is the major cause of drug-induced liver injury and acute liver failure. Approximately 10% of APAP is metabolized by cytochrome P450 (CYP2E1) into toxic *N*-acetyl-p-benzoquinone imine (NAPQI). CYP2E1 also contributes to ethanol metabolism, especially during conditions of high blood ethanol concentration. Acute and chronic ethanol consumption appears to have opposite effects on APAP-induced liver injury. We determined the effects of different doses, pre- and post-treatment, and various schedules of ethanol exposure in APAP-induced liver injury. Treatment with ethanol (0.5 g/kg) after 1 h of APAP (300 mg/kg) administration decreased serum ALT levels, histopathological features, and inflammatory cell infiltration. Moreover, ethanol treatment 1 h after APAP treatment reduced APAP-induced liver injury compared with later administration. Interestingly, ethanol pretreatment did not provide any protective effect. Furthermore, ethanol treatment was associated with a significant decrease in ERK and AKT phosphorylation during the acute injury phase. Ethanol exposure also increased CYP2E1 expression and decreased PCNA expression during the liver regeneration phase.

## 1. Introduction

Acetaminophen (*N*-acetyl-p-aminophenol or paracetamol; APAP) is commonly used as an antipyretic and analgesic drug worldwide [1]. Although APAP is widely regarded as safe and effective at the recommended doses (1–4 g/day), an overdose may cause acute liver injury, fulminant liver failure, and even death [2,3]. At recommended therapeutic doses, the majority (85–90%) of APAP is conjugated with sulfate and glucuronide to form non-toxic metabolites, which are then eliminated from the body [4]. The remaining APAP (approximate 10%) is metabolized by hepatic cytochrome P450 (CYP) enzymes, particularly CYP2E1, to a toxic reactive intermediate, *N*-acetyl-p-benzoquinone imine (NAPQI), which is quickly conjugated to hepatic glutathione (GSH) to produce a non-toxic compound [1,5]. However, at toxic doses, the excessive production of NAPQI results in the depletion of GSH, and NAPQI covalently binds to cellular proteins. This causes mitochondrial dysfunction, increased reactive oxygen species (ROS) production, DNA fragmentation, and eventually hepatocyte cell death [6,7,8].

Ethanol (ethyl alcohol; EtOH) has been one of the most commonly used substances in many cultures for centuries. Ethanol is metabolized primarily by alcohol dehydrogenase (ADH) to form acetaldehyde in the cytosol [9,10]. In addition, CYP family members in the endoplasmic reticulum also contribute to approximately 10% of ethanol oxidative metabolism [9,11]. However, at high ethanol blood concentrations, CYP2E1 plays an important role in oxidizing ethanol to acetaldehyde [12,13]. The metabolism of ethanol to acetaldehyde is accompanied by ROS production, which contributes to the toxicity of ethanol [11,14]. Moreover, acetaldehyde is further metabolized by aldehyde dehydrogenase (ALDH) to acetate in the mitochondria [10].

The interaction between ethanol and APAP is complex. As mentioned above, CYP2E1 is the microsomal enzyme responsible for the metabolism of both ethanol and APAP. Moreover, chronic ethanol intake may enhance APAP-induced liver injury through the upregulation of CYP2E1 activity, increased CYP2E1 synthesis, and decreased GSH levels [15,16,17]. Previous studies indicate that chronic ethanol consumers are at increased risk of APAP-induced hepatotoxicity following repeated overdoses [18,19]. However, therapeutic doses or a single overdose of APAP is not associated with APAP liver injury in chronic alcoholics [20,21].

In contrast, acute ethanol consumption may actually provide protective effects against APAP hepatotoxicity because ethanol is a competitive substrate of CYP2E1. This reduces the level of NAPQI toxic intermediates derived from APAP metabolism [22]. Previous studies have shown that acute ethanol administration attenuates APAP-induced hepatotoxicity in mice when ethanol is administered simultaneously with APAP [23]. A retrospective observational study of approximately 360 patients also demonstrated that acute ethanol ingestion around the same time as APAP overdose is associated with a lower risk of APAP-induced hepatotoxicity [24]. However, the optimal timing and dose of ethanol have not been established. The underlying mechanisms for the effect of ethanol on APAP-induced hepatotoxicity are still unclear. Therefore, we investigated the effects and mechanisms of ethanol in APAP-induced liver injury. We also evaluated the effects of ethanol on liver regeneration following APAP-induced liver injury.

## 2. Materials and Methods

### 2.1. Animals

Adult male C57BL/6C (B6) mice (20–24 g, 8–10 weeks old) were purchased from BioLASCO Taiwan Co., Ltd. (Taipei, Taiwan). All mice were housed in a controlled environment with a 12 h light and dark cycle with ad libitum access to food and water. All animal experimental procedures were conducted in accordance with the guidelines of the Animal Welfare Act and Guide for Care and Use of Laboratory Animals from the National Institute of Health. The protocols were approved by the Institutional Animal Care and Use Committee at the Chang Gung Memorial Hospital.

### 2.2. Experimental Model and Treatments

Initial studies evaluated the effects of different doses of ethanol. The mice were randomly assigned into 8 groups (*n* = 6/group). Acetaminophen (Sigma Chemical Co., St. Louis, MO, USA) was dissolved in normal saline at a concentration of 20 mg/mL. Of the 8 groups, 6 groups received an intraperitoneal hepatotoxic injection of acetaminophen (300 mg/kg) and 1 h later the mice were intraperitoneally [25] treated with ethanol at a concentration of 0, 0.1, 0.25, 0.5, 1, or 2 g/kg [26]. The other 2 control groups received an equal volume of normal saline intraperitoneally and were treated with an equal volume of PBS or ethanol (0.5 g/kg) 1 h later. After 16 h, all of the animals were sacrificed by cervical dislocation under isoflurane anesthesia. Blood and liver samples were obtained for further analysis.

Next, we examined the effects of ethanol pre- and post-treatment. The mice were randomly assigned to 5 groups (*n* = 6/group). Of the 5 groups, 3 groups received an intraperitoneal hepatotoxic injection of acetaminophen (300 mg/kg), and then 2 groups were injected with ethanol (0.5 g/kg) intraperitoneally before or after 1 h of APAP administration. The other 2 control groups received an equal volume of normal saline intraperitoneally and were then treated with an equal volume of PBS or ethanol (0.5 g/kg). After 16 h, all of the animals were sacrificed.

We further examined the effects of the ethanol dose schedule. The mice were randomly assigned into 6 groups (*n* = 6/group). Four of the groups received an intraperitoneal hepatotoxic injection of acetaminophen (300 mg/kg). The mice then received an intraperitoneal injection of ethanol (0.5 g/kg) at 1, 2, and 4 h after APAP administration. The other 2 control groups were administered an equal volume of normal saline intraperitoneally followed by an equal volume of PBS or ethanol (0.5 g/kg). After 16 h, all of the animals were sacrificed.

Finally, we compared the liver regeneration profiles of the APAP and ethanol treatment groups over a time course of 16 to 72 h. Two groups of animals were sacrificed at 16, 48, and 72 h following APAP administration.

### 2.3. Measurement of Alanine Transaminase Levels in Serum

Blood samples were collected by cardiac puncture, stored at room temperature for 1 h, and centrifuged at 12,000× *g* twice for 5 min each. Serum alanine transaminase (ALT) levels were measured using the VITROS DT60 II Chemistry System (Ortho-Clinical Diagnostics, Raritan, NJ, USA).

### 2.4. Measurement of GSH Levels in the Liver

The liver tissues were homogenized in cold Tris-HCl buffer on ice. The homogenates were centrifuged at 10,000× *g* for 15 min at 4 °C and the supernatants were deproteinated and measured for GSH levels according to the manufacturer’s instructions for the Glutathione assay kit (Cayman Chemical Co., Ann Arbor, MI, USA).

### 2.5. Histology and Immunohistochemistry

The left lobes of the liver were harvested and fixed in 4% paraformaldehyde and embedded in paraffin. Sections of 4 µm thickness were stained with hematoxylin and eosin (H&E) for histological examination.

For immunohistochemical staining, the sections were incubated with primary antibody against Ly6G (neutrophil; 1:500; BD Biosciences Pharmingen, San Diego, CA, USA) or Mac-2 (macrophage; 1:500; eBioscience, Inc., San Diego, CA, USA). After rinsing in PBS, the sections were incubated with a biotin-conjugated secondary antibody (IHC Select; Merck Millipore, Burlington, MA, USA) for 1 h. Finally, a peroxidase reaction was performed following the manufacturer’s protocol (IHC Select; Millipore). To assess cell proliferation, the sections were stained with proliferating cell nuclear antigen (PCNA) (Cell Signaling Technology, Danvers, MA, USA).

### 2.6. Measurement of TNF-α and IL-6 in the Liver

We measured the levels of proinflammatory cytokines including tumor necrosis factor (TNF)-α and interleukin (IL)-6 in the liver. The liver tissues were homogenized in lysis buffer (20 mM HEPES, 3 mM MgCl_2_, 1 mM 2-mercaptoethanol [2-ME], 150 mM NaCl, 1 M dithiothreitol [DTT], 0.25 μg leupeptin, 0.1 mM phenyl-methyl-sulfonyl fluoride [PMSF], 0.05 μg pepstatin A, and 0.01 μg aprotinin) on ice. The homogenates were centrifuged at 12,000× *g* for 10 min at 4 °C and the supernatants were used for the measurement of cytokine levels according to the manufacturer’s instructions. Finally, the absorbance was read at 450 nm.

### 2.7. Western Blotting

The liver tissues were homogenized in lysis buffer, sonicated for 15 s, and centrifuged at 12,000× *g* for 10 min at 4 °C. Equal amounts of protein were separated on 10% sodium dodecyl sulfate polyacrylamide gels by electrophoresis and then transferred to polyvinylidene fluoride membranes. After blocking in 5% fat-free milk solution for 1 h and rinsing 3 times with Tris-buffer (1% Tween-20), the membranes were incubated with specific primary antibodies against extracellular signal-regulated kinase (ERK), c-Jun N-terminal kinase (JNK), protein kinase B (AKT), phospho-ERK, phospho-JNK, phospho-AKT, CYP2E1, and PCNA (1:1000; Cell Signaling Technology, Danvers, MA, USA) overnight at 4 °C. Following a washing step and incubation with horseradish peroxidase-conjugated secondary antibody (Cell Signaling Technology, Danvers, MA, USA) for 1 h at room temperature, the immune complexes were visualized using an enhanced chemiluminescence system. The antibody specific to β-actin (Proteintech Group, Inc., Chicago, IL, USA) was used to confirm equal loading.

### 2.8. Statistical Analysis

All data are presented as the mean ± standard error of mean (SEM) (*n* = 5–6 mice for each group). One-way analysis of variance (ANOVA) and Tukey’s multiple comparison tests were used to analyze the results. Prism 6.0 Software (GraphPad Software Inc., San Diego, CA, USA) was used for statistical analyses. *p* < 0.05 was considered statistically significant.

## 3. Results

### 3.1. Effects of Different Doses of Ethanol on Serum ALT Levels in APAP-Induced Liver Injury

**Figure 1:**
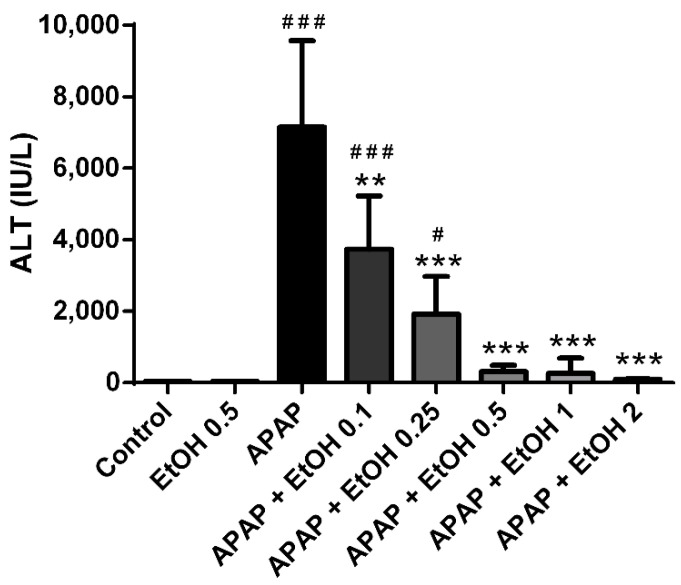
The effects of different ethanol doses on serum ALT levels in acetaminophen-induced liver injury. Mice were administrated APAP (300 mg/kg) or an equal volume of saline (control) intraperitoneally and treated with various concentrations of ethanol (0, 0.1, 0.25, 0.5, 1 or 2 g/kg) after 1 h. Mice were sacrificed after 16 h of APAP challenge. Each value represents the mean ± SEM; *n* = 6 for each group. ^#^ *p* < 0.05, ^###^ *p* < 0.005 vs. control group; ** *p* < 0.01, *** *p* < 0.005 vs. APAP group.

To investigate the effects of different doses of ethanol in APAP-induced liver injury, we measured ALT levels of the serum following APAP-induced liver injury. The serum ALT levels were markedly elevated in the APAP (300 mg/kg) group compared with the control group (*p* < 0.005). No differences were observed in the serum ALT levels between the control and ethanol (0.5 g/kg) alone groups. After 1 h of APAP administration, treatment with ethanol (0.1, 0.25, 0.5, 1, and 2 g/kg) significantly attenuated serum ALT levels compared with the APAP group (*p* < 0.01, 0.005, 0.005, 0.005, and 0.005, respectively). However, extreme low dose ethanol (0.1 and 0.25 g/kg) treatment groups still exhibited significantly elevated serum ALT levels compared the control group (*p* < 0.005 and 0.05, respectively). Moreover, when treated with low to moderate doses of ethanol (0.5, 1, and 2 g/kg), the serum ALT levels did not increase significantly compared with the control group (Figure 1). These results indicate that treatment with ethanol can ameliorate APAP-induced liver injury and 0.5 g/kg ethanol administration following 1 h of APAP challenge is the minimal dose for the best protective effect.

### 3.2. Effect of Ethanol Pre- and Post-Treatment on Serum ALT Levels, Hepatic GSH Levels, and Hepatic Histological Changes

**Figure 2:**
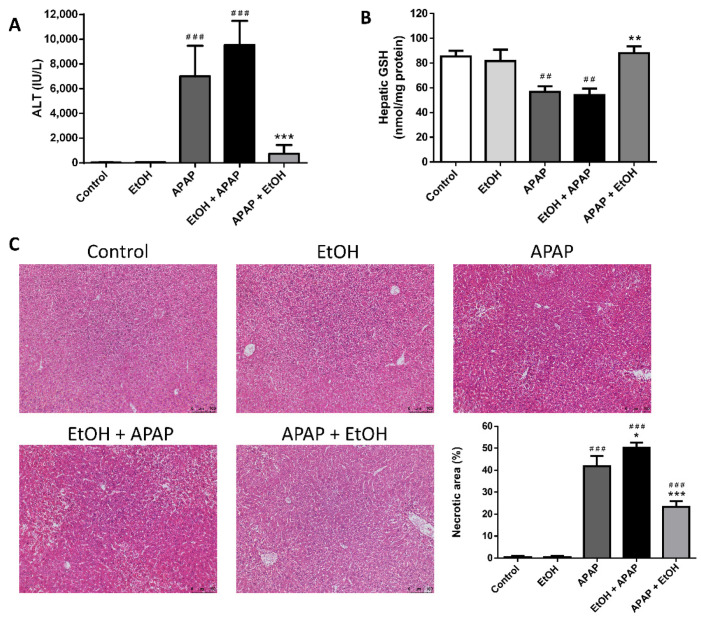
The effects of the time of ethanol administration on serum ALT levels (**A**), hepatic GSH levels (**B**), and histological changes (**C**) during acetaminophen-induced liver injury. Mice were administrated APAP (300 mg/kg) or an equal volume of saline (control) intraperitoneally and treated with 0.5 g/kg of ethanol before and after 1 h. Mice were sacrificed after 16 h of APAP challenge. (**A**,**B**) Each value represents the mean ± SEM; *n* = 6 for each group. ^##^ *p* < 0.01, ^###^ *p* < 0.005 vs. control group; ** *p* < 0.01, *** *p* < 0.005 vs. APAP group. (**C**) Representative histological changes of the liver tissues obtained from different groups. (100× magnifications are shown). Quantification of the percentage of necrotic areas. Each value represents the mean ± SEM; *n* = 6 for each group. ^###^ *p* < 0.005 vs. control group; * *p* < 0.05, *** *p* < 0.005 vs. APAP group.

The APAP and ethanol pre-treatment group had significantly elevated serum ALT levels compared with the control group (*p* < 0.005 and 0.005, respectively); however, there were no differences in the serum ALT levels between the control and ethanol alone groups. Pretreatment with ethanol (0.5 g/kg) 1 h before APAP administration did not significantly alter serum ALT levels compared with the APAP group. Interestingly, ethanol post-treatment (0.5 g/kg) 1 h after APAP challenge significantly attenuated serum ALT levels compared with the APAP group (*p* < 0.005) (Figure 2A).

There were no differences in the hepatic GSH levels among the control, ethanol alone, and ethanol post-treatment groups. The APAP and ethanol pre-treatment groups had significantly lowered hepatic GSH levels compared with the control group (*p* < 0.01 and 0.01, respectively). There was also no significant difference in the hepatic GSH levels between the APAP and ethanol pre-treatment groups. Moreover, posttreatment with ethanol (0.5 g/kg) 1 h after APAP administration significantly elevated hepatic GSH levels compared with the APAP group (*p* < 0.01) (Figure 2B).

Histopathological examination demonstrated severe sinusoidal congestion and centrilobular necrosis in the APAP and ethanol pre-treatment groups. The histological appearance of the liver tissues in the control and ethanol alone groups were considered normal. Consistent with the serum ALT level results, post-treatment with ethanol (0.5 g/kg) 1 h after APAP administration markedly altered pathological features following APAP-induced liver injury (Figure 2C). Taken together, our results suggest that only ethanol post-treatment, but not ethanol pre-treatment, attenuates APAP-induced liver injury.

### 3.3. Effects of Ethanol Pretreatment and Posttreatment on the Infiltration of Neutrophil and Macrophage in the Liver

**Figure 3:**
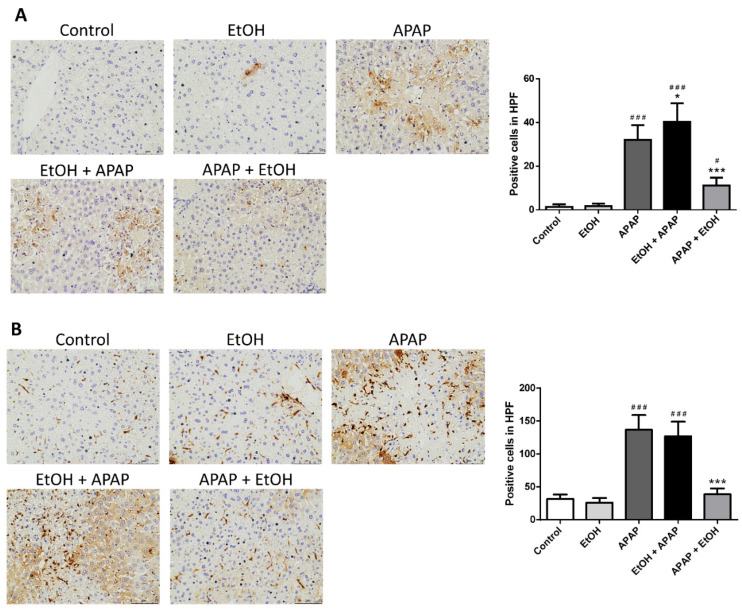
The effects of timing of ethanol treatment on neutrophil (**A**) and macrophage (**B**) infiltration in acetaminophen-induced liver injury. Mice were administrated APAP (300 mg/kg) or an equal volume of saline (control) intraperitoneally and treated with 0.5 g/kg of ethanol before and after 1 h. Mice were sacrificed after 16 h of APAP challenge. The liver sections were immunostained with (**A**) Ly6G or (**B**) Mac-2 antibody (brown). Representative immunohistochemical staining of the liver tissues obtained from different groups. (200× magnifications are shown). Quantification of positive inflammatory cells was analyzed under high power field (HPF). Each value represents the mean ± SEM; *n* = 6 for each group. ^#^ *p* < 0.05, ^###^ *p* < 0.005 vs. control group; * *p* < 0.05, *** *p* < 0.005 vs. APAP group.

Immunohistochemical staining with Ly6G antibody, a reliable marker for identifying granulocytes, was used to determine inflammatory infiltration of neutrophils during APAP-induced liver injury. The APAP and ethanol pretreatment group displayed abundant infiltrated neutrophils in the hepatic injured area compared with the control group. Moreover, ethanol post-treatment (0.5 g/kg) 1 h after APAP challenge significantly decreased neutrophil infiltration in the liver parenchyma following APAP-induced liver injury (Figure 3A).

Immunohistochemical staining with Mac-2 antibody, a macrophage cell surface marker, was used to assess the infiltration and accumulation of macrophages following APAP-induced liver injury. The APAP and ethanol pre-treatment group showed a marked infiltration of macrophages in the hepatic injured area compared with the control group. Furthermore, post-treatment with ethanol (0.5 g/kg) 1 h after APAP challenge significantly reduced macrophage infiltration and accumulation in the liver parenchyma following APAP-induced liver injury (Figure 3B).

### 3.4. Effects of Ethanol Pre- and Post-Treatment on Hepatic TNF-α and IL-6 Levels

**Figure 4:**
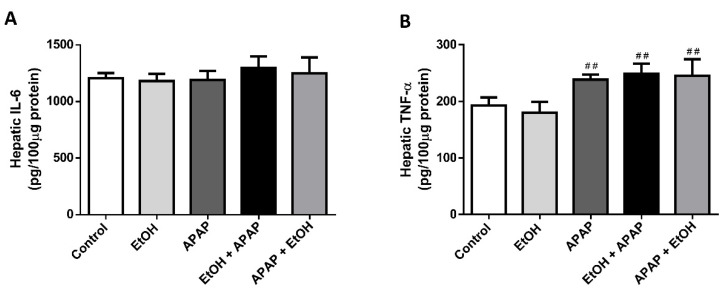
The effects of timing of ethanol treatment on hepatic IL-6 (**A**) and TNF-α (**B**) levels in acetaminophen-induced liver injury. Mice were administrated APAP (300 mg/kg) or an equal volume of saline (control) intraperitoneally and treated with 0.5 g/kg of ethanol before and after 1 h. Mice were sacrificed after 16 h of APAP challenge. Each value represents the mean ± SEM; *n* = 6 for each group. ^##^ *p* < 0.01 vs. control group.

To investigate the effects of ethanol pre- and post-treatment on proinflammatory cytokine expression during APAP-induced liver injury, we assessed the levels of TNF-α and IL-6 in the liver tissue using ELISA. There were no differences in hepatic IL-6 levels among all the groups. In addition, APAP, ethanol pre-treatment, and ethanol post-treatment groups exhibited significantly increased hepatic TNF-α levels compared with the control group (*p* < 0.01, 0.01, and 0.01, respectively). However, there were no differences in the hepatic TNF-α levels among the APAP, ethanol pre-treatment, and ethanol post-treatment groups (Figure 4). These results suggest that although post-treatment with ethanol may attenuate APAP-induced liver injury, these proinflammatory cytokines do not appear to be involved in the protective mechanism.

### 3.5. Effects of Ethanol Pre- and Post-Treatment on ERK, JNK, and AKT Expression and Phosphorylation in the Liver

**Figure 5:**
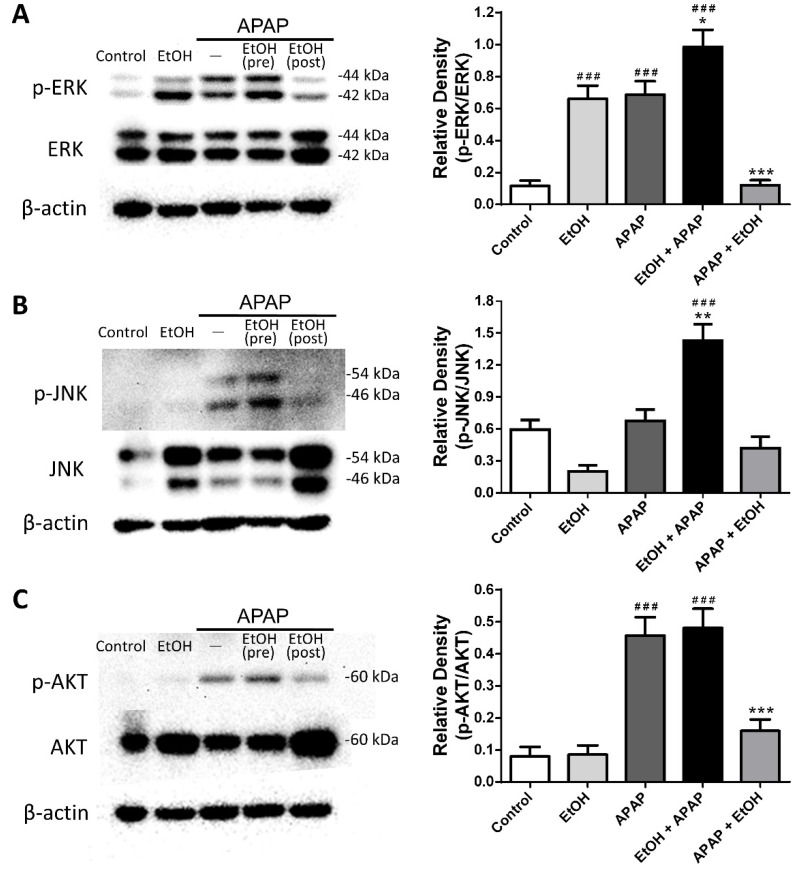
The effects of timing of ethanol treatment on hepatic ERK (**A**), JNK (**B**), and AKT (**C**) expression and phosphorylation in acetaminophen-induced liver injury. Mice were administrated APAP (300 mg/kg) or an equal volume of saline (control) intraperitoneally and treated with 0.5 g/kg of ethanol before and after 1 h. Mice were sacrificed after 16 h of APAP challenge. The bands were analyzed using densitometry. Each value represents the mean ± SEM; *n* = 5–6 for each group. ^###^ *p* < 0.005 vs. control group; * *p* < 0.05, ** *p* < 0.01, *** *p* < 0.005 vs. APAP group. Uncropped western blots from Figure 5 were shown in Supplementary Materials (Figure S1–S3).

We further examined hepatic ERK, JNK, and AKT protein expression and phosphorylation during APAP-induced liver injury. Phospho-ERK expression was significantly increased in the ethanol alone, APAP, and ethanol pre-treatment groups compared with the control group. Post-treatment with ethanol (0.5 g/kg) 1 h after APAP challenge markedly reduced phospho-ERK expression; however, ethanol pretreatment appeared to increase phospho-ERK expression compared with APAP group (Figure 5A). Moreover, there were no significant differences in hepatic phospho-JNK levels among the control, ethanol alone, APAP, and ethanol post-treatment groups. Only the ethanol pre-treatment group exhibited significantly increased phospho-JNK expression compared with the control or APAP group (Figure 5B).

Furthermore, no significant difference in hepatic phospho-AKT expression was observed between the control and ethanol alone groups. Phospho-AKT expression was markedly increased in the APAP and ethanol pre-treatment groups compared with the control group. Post-treatment with ethanol (0.5 g/kg) 1 h after APAP challenge decreased phospho-AKT protein expression (Figure 5C). Taken together, our results demonstrate that although ethanol alone can induce phospho-ERK expression, post-treatment with ethanol may attenuate both ERK and AKT phosphorylation and activation in the liver following APAP-induced liver injury.

### 3.6. Effects of Different Schedules of Ethanol Treatment on APAP-Induced Liver Injury

**Figure 6:**
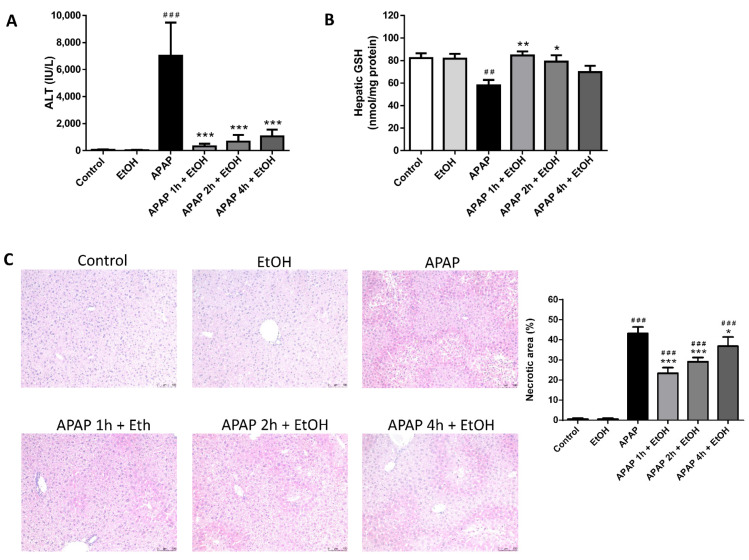
The effects of different ethanol administration times on serum ALT levels (**A**), hepatic GSH levels (**B**), and histological changes (**C**) in acetaminophen-induced liver injury. Mice were administrated APAP (300 mg/kg) or an equal volume of saline (control) intraperitoneally and treated with 0.5 g/kg of ethanol after 1, 2, or 4 h. Mice were sacrificed after 16 h of APAP challenge. (**A**,**B**) Each value represents the mean ± SEM; *n* = 6 for each group. ^##^ *p* < 0.01, ^###^ *p* < 0.005 vs. control group; * *p* < 0.05, ** *p* < 0.01, *** *p* < 0.005 vs. APAP group. (**C**) Representative histological changes of the liver tissues obtained from different groups. (100× magnifications are shown). Quantification of the percentage of necrotic areas. Each value represents the mean ± SEM; *n* = 6 for each group. ^###^ *p* < 0.005 vs. control group; * *p* < 0.05, *** *p* < 0.005 vs. APAP group.

To investigate the effect of the timing of ethanol administration on APAP-induced liver injury, ethanol was injected at 1, 2, or 4 h after APAP administration. Serum ALT levels were significantly increased in the APAP (300 mg/kg) group compared with the control group (*p* < 0.005). All three ethanol treatment groups exhibited decreased serum ALT levels compared with the APAP group (*p* < 0.005, 0.005, and 0.005, respectively). Moreover, ethanol treatment 1 h after APAP was the most effective at lowering serum ALT levels (*p* < 0.05 compared with 4 h after APAP administration) (Figure 6A). In addition, there was no significant difference in ethanol administered 1 or 2 h after APAP challenge and no significant difference in ethanol given 2 or 4 h after APAP challenge.

Hepatic GSH levels were significantly decreased in the APAP group compared with the control group (*p* < 0.01). Ethanol treatment 1 and 2 h after APAP administration significantly elevated hepatic GSH levels compared with the APAP group (*p* < 0.01 and 0.05, respectively). However, there was no significant difference in the hepatic GSH levels between the APAP and ethanol treatment 4 h after APAP groups (Figure 6B).

Histopathological examination results were consistent with the findings of serum ALT levels. Severe sinusoidal congestion and centrilobular necrosis were observed in the APAP group. All three ethanol treatment groups exhibited less of these features following APAP-induced liver injury. Furthermore, ethanol treatment at 1 h after APAP was most effective at reducing necrosis compared with the other ethanol treatment groups (2 h or 4 h after APAP) (Figure 6C).

**Figure 7:**
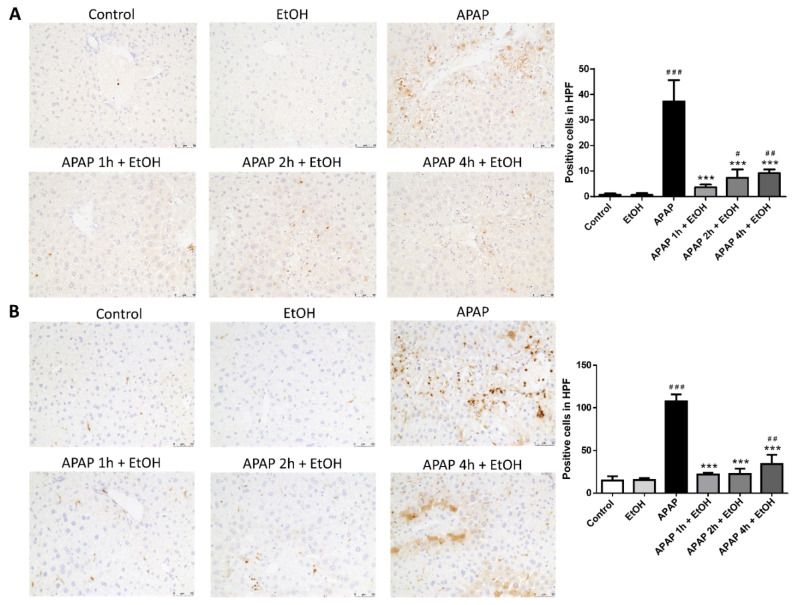
The effects of different ethanol administration times on neutrophil (**A**) and macrophages (**B**) infiltration in acetaminophen-induced liver injury. Mice were administrated APAP (300 mg/kg) or an equal volume of saline (control) intraperitoneally and treated with 0.5 g/kg of ethanol after 1, 2, or 4 h. Mice were sacrificed after 16 h of APAP challenge. The liver sections were immunostained with (**A**) Ly6G or (**B**) Mac-2 antibody (brown). Representative immunohistochemical staining of the liver tissues obtained from different groups. (200× magnifications are shown). Quantification of positive inflammatory cells was analyzed under HPF. Each value represents the mean ± SEM; *n* = 6 for each group. ^#^ *p* < 0.05, ^##^ *p* < 0.01, ^###^ *p* < 0.005 vs. control group; *** *p* < 0.005 vs. APAP group.

We performed immunohistochemical staining with Ly6G and Mac-2 antibodies following APAP-induced liver injury. Significant neutrophil and macrophage infiltration in the liver parenchyma was observed in the APAP group. All three ethanol treatment groups showed a reduced hepatic neutrophil (Figure 7A) and macrophage (Figure 7B) infiltration compared with the APAP group. Moreover, ethanol treatment 1 h after APAP administration was most effective at reducing neutrophil and macrophage infiltration compared with the other ethanol treatment groups (2 h or 4 h after APAP) did. Taken together, these results demonstrate that treatment with ethanol 1, 2, or 4 h after APAP administration attenuated APAP-induced liver injury, including serum ALT levels, histological features, and inflammatory cell infiltration. However, ethanol treatment 1 h after APAP challenge provided the best protective effect following APAP-induced liver injury.

### 3.7. Effects of Ethanol Treatment on Liver Regeneration

**Figure 8:**
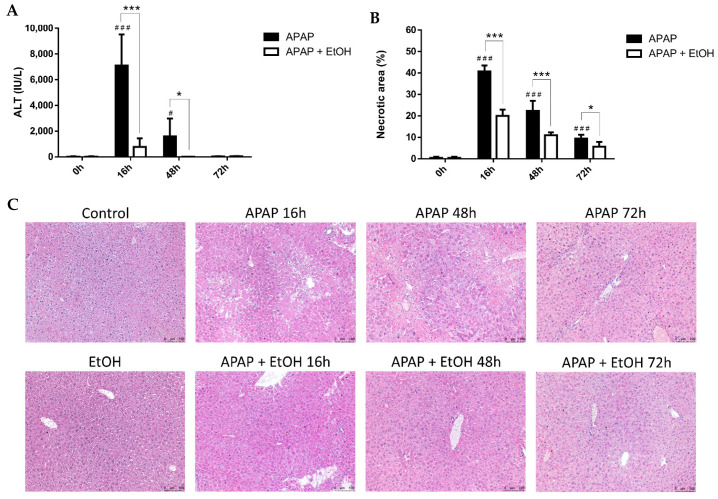
The effects of ethanol on serum ALT levels (**A**) and histological changes (**B**) in acetaminophen-induced liver injury after 16, 48, and 72 h. Mice were administrated APAP (300 mg/kg) or an equal volume of saline (control) intraperitoneally and treated with 0.5 g/kg of ethanol after 1 h. Mice were sacrificed after 16, 48, or 72 h of APAP challenge. (**A**) Each value represents the mean ± SEM; *n* = 6 for each group. ^#^ *p* < 0.05, ^###^ *p* < 0.005 vs. control group; * *p* < 0.05, *** *p* < 0.005 vs. APAP group. (**C**) Representative histological changes of the liver tissues obtained from different groups. (100× magnifications are shown). Quantification of the percentage of necrotic areas. Each value represents the mean ± SEM; *n* = 6 for each group. ^###^ *p* < 0.005 vs. control group; * *p* < 0.05, *** *p* < 0.005 vs. APAP group.

To understand the effects of ethanol treatment on liver regeneration following APAP-induced liver injury, we compared serum ALT levels and histopathological appearance in the APAP and ethanol treatment groups at 16, 48, and 72 h (Figure 8A,B). Serum ALT levels were markedly elevated and peaked at 16 h after APAP administration in the APAP group. Afterwards, serum ALT levels gradually decreased after 48 h of APAP administration in the APAP and ethanol treatment groups. Moreover, serum ALT levels were significantly reduced at 16 and 48 h after APAP challenge in the ethanol treatment group compared with the APAP group (Figure 8A). Histopathology revealed a similar pattern with serum ALT levels, in which the most severe necrosis was observed at 16 h after APAP challenge, and a gradual recovery after 48 h in the APAP group. Ethanol treatment significantly diminished the necrotic area over time compared with the control group (Figure 8B).

**Figure 9:**
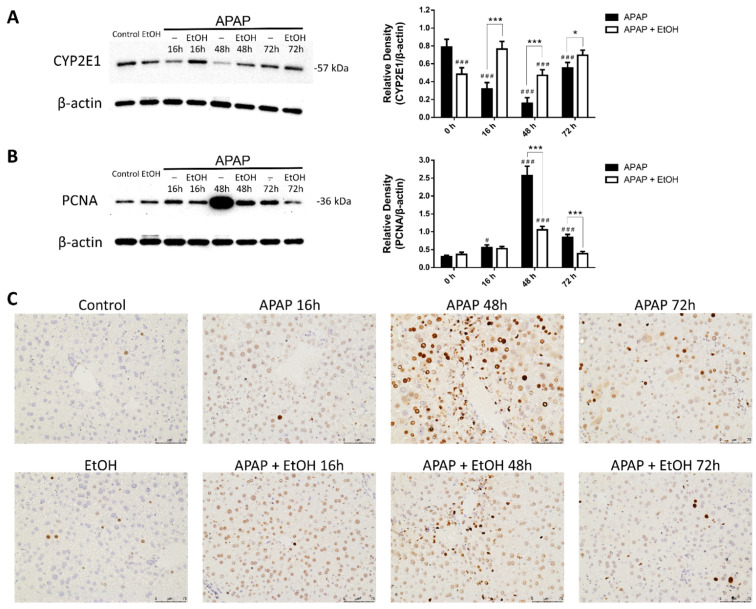
The effects of ethanol on hepatic CYP2E1 (**A**) and PCNA (**B**,**C**) expression in acetaminophen-induced liver injury after 16, 48, and 72 h. Mice were administrated APAP (300 mg/kg) or an equal volume of saline (control) intraperitoneally and treated with 0.5 g/kg of ethanol after 1 h. Mice were sacrificed after 16, 48, or 72 h of APAP challenge and liver tissues were harvested. (**A**,**B**) The bands were analyzed using densitometry. Each value represents the mean ± SEM; *n* = 5–6 for each group. ^#^ *p* < 0.05, ^###^ *p* < 0.005 vs. control group; * *p* < 0.05, *** *p* < 0.005 vs. APAP group. (**C**) The liver sections were immunostained with PCNA antibody (brown). Representative immunohistochemical staining of the liver tissues obtained from different groups. (200× magnifications are shown). Uncropped western blots from Figure 9 were shown in Supplementary Materials (Figure S4–S5).

We analyzed hepatic CYP2E1 expression over the time course from 16 to 72 h following APAP-induced liver injury by western blot analysis. In the APAP group, CYP2E1 expression significantly decreased at 16 h and its expression was the lowest at 48 h; thereafter, it appeared to return to normal at 72 h. Interestingly, although the ethanol alone group showed significantly decreased CYP2E1 expression compared with the control group, there was increased CYP2E1 expression at 16, 48, and 72 h after APAP administration in the ethanol treatment group compared with the APAP group (*p* < 0.005, 0.005, and 0.05, respectively, Figure 9A).

We also evaluated the expression of hepatic PCNA, a cell regeneration marker, over the time course from 16 to 72 h following APAP-induced liver injury by western blot analysis and immunohistochemistry. In the APAP group, PCNA expression was slightly increased at 16 h, peaked at 48 h, and gradually decreased at 72 h following APAP administration (*p* < 0.005, 0.005, and 0.05, respectively, Figure 9B). No significant difference was observed at 16 h after APAP challenge between the APAP and ethanol treatment groups. Moreover, there was a significant decrease in PCNA expression at 48 and 72 h after APAP administration in the ethanol treatment group compared with the APAP group (*p* < 0.005 and 0.005, respectively, Figure 9B). The immunohistochemical staining for PCNA showed a similar trend with the results of western blot analysis (Figure 9C). Taken together, these results indicate that treatment with ethanol significantly increased CYP2E1 expression and decreased PCNA expression compared with the APAP group.

## 4. Discussion

In the present study, we investigated the effects of different doses, pre-treatment or post-treatment, and different schedules of ethanol administration in APAP-induced liver injury. After APAP (300 mg/kg) administration for 16 h to induce liver injury, serum ALT levels were markedly increased. Treatment with ethanol after 1 h of APAP administration decreased serum ALT levels, necrotic areas, and inflammatory cell infiltration. Moreover, 0.5 g/kg ethanol was the minimal dose for the greatest protective effect. Ethanol treatment 1 h after APAP resulted in superior effects with respect to reducing APAP-induced liver injury compared with administering later. Interestingly, ethanol (0.5 g/kg) pre-treatment did not provide any protection against APAP-induced liver injury. Furthermore, ethanol treatment was significantly associated with decreased ERK and AKT phosphorylation in the acute liver injury phase. Ethanol treatment also increased CYP2E1 expression and decreased PCNA expression during the liver regeneration phase.

It is well established that innate immunity and the inflammatory response have a vital role in the amplification and injury phase during APAP-induced liver injury [27,28]. At toxic doses of APAP, excess NAPQI depletes GSH and forms protein adducts, which leads to mitochondrial dysfunction, oxidative stress, DNA fragmentation, and hepatocyte necrosis [1,6]. Damage-associated molecular patterns (DAMPs), released during necrotic hepatocyte death, are recognized by resident macrophages (Kupffer cells, KC) via toll-like receptors and subsequently cause their activation [29,30,31,32]. The activated KCs secrete numerous chemokines, which facilitate the infiltration of neutrophils into the liver, and cytokines, which amplify the inflammatory response and further aggravate liver damage [33,34]. In the current study, ethanol treatment attenuated APAP-induced liver injury, accompanied by a significant decrease in the infiltration of inflammatory cells compared with the APAP group. The protective effects of ethanol on APAP-induced liver injury may be responsible for reduced activation of macrophages and decreased neutrophil infiltration. However, our data show that there were no differences in hepatic proinflammatory cytokine levels among the APAP, ethanol pre-treatment, and ethanol post-treatment groups. The protective effects of pre-treatment with ethanol may not be associated with altered proinflammatory cytokine levels.

The ERK, one of the MAPK family, is an essential signaling pathway regulating numerous cellular processes, including the inflammatory process, oxidative stress, apoptosis, proliferation, and differentiation [35]. A previous study has demonstrated that ERK signaling contributes to the oxidative signaling pathway and plays an important role in APAP-induced hepatotoxicity, whereas the inhibition of ERK provides protective effects against such oxidative disorders [36]. AKT, an another important signaling pathway, plays a pivotal role in various cellular processes, such as cell survival, proliferation, apoptosis, and inflammatory responses against extracellular stimuli [37]. Moreover, recent studies have revealed that the inhibition of the AKT signaling attenuated APAP-induced liver injury by inhibiting the activation of apoptotic signaling pathways and regulating survival mechanism [38,39]. In our study, phospho-ERK and phosphor-AKT expression significantly increased in the APAP and ethanol pre-treatment group. Post-treatment with ethanol effectively alleviated both ERK and AKT phosphorylation following APAP-induced liver injury.

Previous studies have demonstrated that acute and chronic ethanol consumption could provide contradictory effects on APAP-induced liver injury [40]. Chronic ethanol consumption may increase APAP-induced hepatotoxicity by enhancing CYP2E1 activity and decreasing GSH stores [16,17]. However, acute ethanol ingestion may protect against APAP-induced hepatotoxicity through competition with APAP for CYP2E1 and the timing of ethanol treatment related to APAP administration appears to be important [22,23]. In the present study, treatment with ethanol 1 h after APAP administration provided a more significant protective effect on APAP-induced hepatotoxicity compared with later treatment. However, pre-treatment with ethanol at 1 h before APAP challenge did not offer any protection against APAP-induced liver injury. A plausible reason for this is that, while ethanol is given before APAP, ethanol itself may result in the induction of CYP2E1, which facilitates APAP metabolism to its toxic byproduct, NAPQI. Moreover, we found that there was increased CYP2E1 expression at 16, 48, and 72 h in the ethanol treatment group compared with the APAP group. The possible cause may be that ethanol treatment, in part, competes with APAP for CYP2E1, which leads to less NAPQI depletion by APAP metabolism. Eventually, more CYP2E1 is preserved in the ethanol treatment group.

PCNA, a classic marker for cell proliferation and tissue regeneration, plays an essential role in DNA replication. A recent study demonstrated that PCNA gradually increased and peaked at 48 h after APAP administration [41], which was consistent with our results. The effects of ethanol on cell proliferation vary depending on the dose, amount, and the duration of ethanol consumption. Previous studies demonstrated that a chronic moderate-dose ethanol diet could inhibit liver regeneration after partial hepatectomy in rats by inhibiting DNA synthesis and altering post-transcriptional levels [42,43]. Moreover, a recent study using human hepatoma HepaRG cells showed that ethanol exposure was accompanied by decreased cell viability, lower protein levels, and reduced DNA synthesis, which resulted in the inhibition of cell proliferation [44]. However, another study demonstrated that light ethanol consumption enhances hepatic regenerative activity after partial hepatectomy in rats [45]. In the present study, our data revealed that treatment with ethanol significantly decreased PCNA expression at 48 and 72 h after APAP administration compared with the APAP group. The protective effects of ethanol treatment may not be associated with increased proliferation or regeneration. One possible reason is that injury itself is a strong stimulus for cell proliferation. The APAP group exhibited more aggravated liver injury and a concomitant increase in cell proliferation activity.

The present study had some potential limitations. Fist, we did not measure the NAPQI levels. Second, we only measured the inflammatory cytokine levels at 16 h after APAP administration. Third, it is not clear whether ERK and AKT play major roles in the protective effect of ethanol treatment or are merely secondary to reduced liver damage. To make clear these concerns, we would add another experimental design and evaluate the application of low-dose ethanol for the treatment of APAP-induced liver injury in our future studies.

## 5. Conclusions

In conclusion, post-treatment with ethanol (0.5 g/kg), but not pre-treatment, after 1 h of APAP administration decreased serum ALT levels, histopathological appearance, and inflammatory cell infiltration. The protective effects of ethanol treatment after 1 h of APAP were superior to later treatment. Moreover, ethanol treatment was associated with decreased ERK and AKT phosphorylation, increased CYP2E1 expression, and decreased PCNA expression. In general, ethanol is extremely accessible in most areas and could be used as a complementary or alternative treatment for APAP-induced liver injury; however, further studies regarding its clinical utilization will be needed.

## Data Availability

The data presented in this study are available on request from the corresponding author.

## Supplementary Materials

The following are available online at https://www.mdpi.com/article/10.3390/life11101094/s1, Figure S1:Uncropped western blots from Figure 5A, Figure S2: Uncropped western blots from Figure 5B, Figure S3. Uncropped western blots from Figure 5C, Figure S4. Uncropped western blots from Figure 9A, Figure S5. Uncropped western blots from Figure 9B.
