# Supplementary material for: The Timing and Effects of Low-Dose Ethanol Treatment on Acetaminophen-Induced Liver Injury
Source: Life (Basel). 2021 Oct 15;11(10):1094. doi: 10.3390/life11101094 (PMC8539755; doi:10.3390/life11101094)
Supplement: Supplementary file 1 [file life-11-01094-s001.zip › life-1377442-supplementary.pdf]

life

Supplementary Materials:

The Timing and Effects of Low-dose Ethanol Treatment on Acetaminophen-induced Liver Injury

Fu-Chao Liu [1,2,†], Huang-Ping Yu [1,2,†], Chia-Chih Liao [1,2], An-Hsun Chou [1,2] and Hung-Chen Lee [1,2,*]

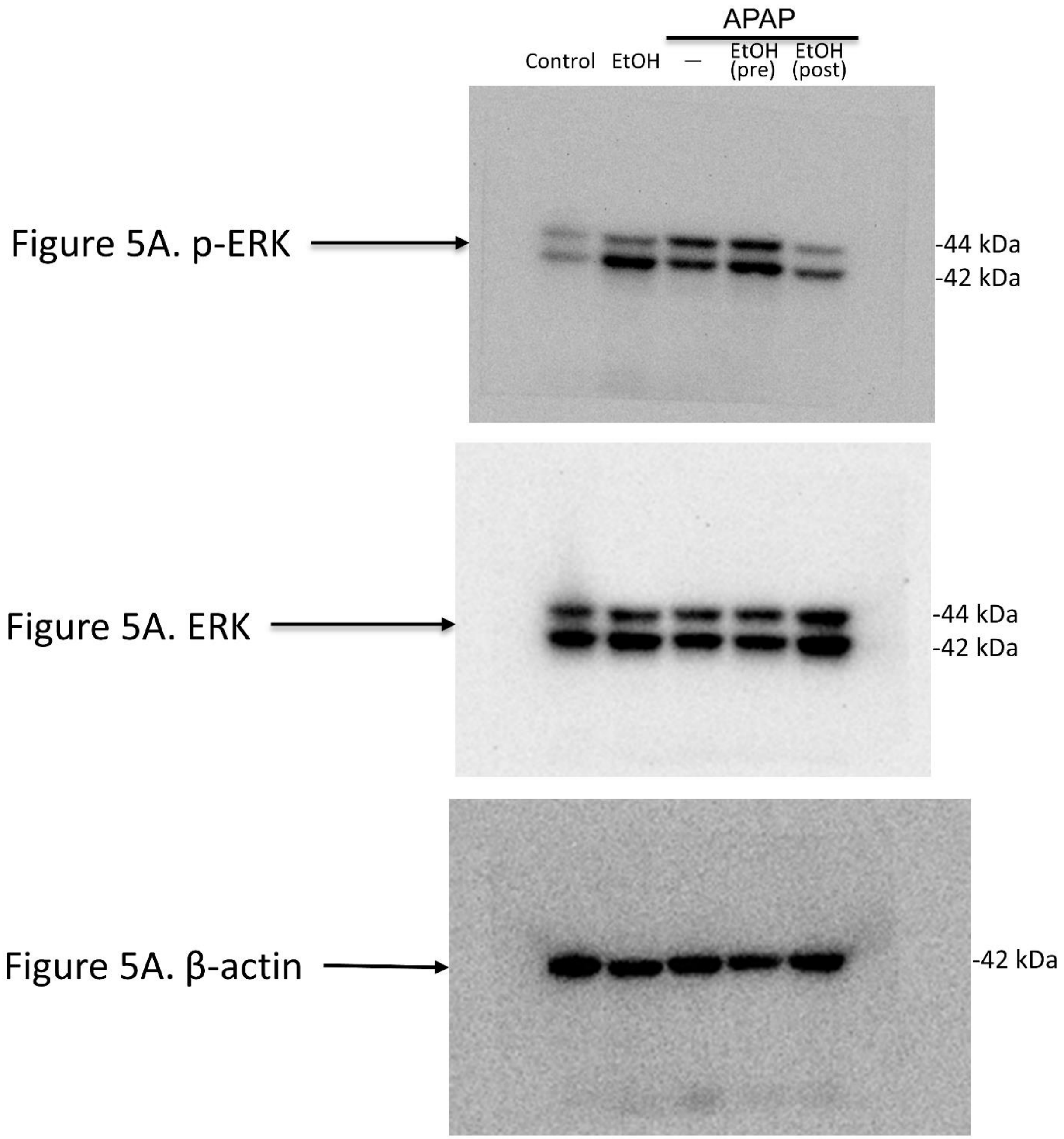

Figure S1. Uncropped Western blots from Figure 5A.

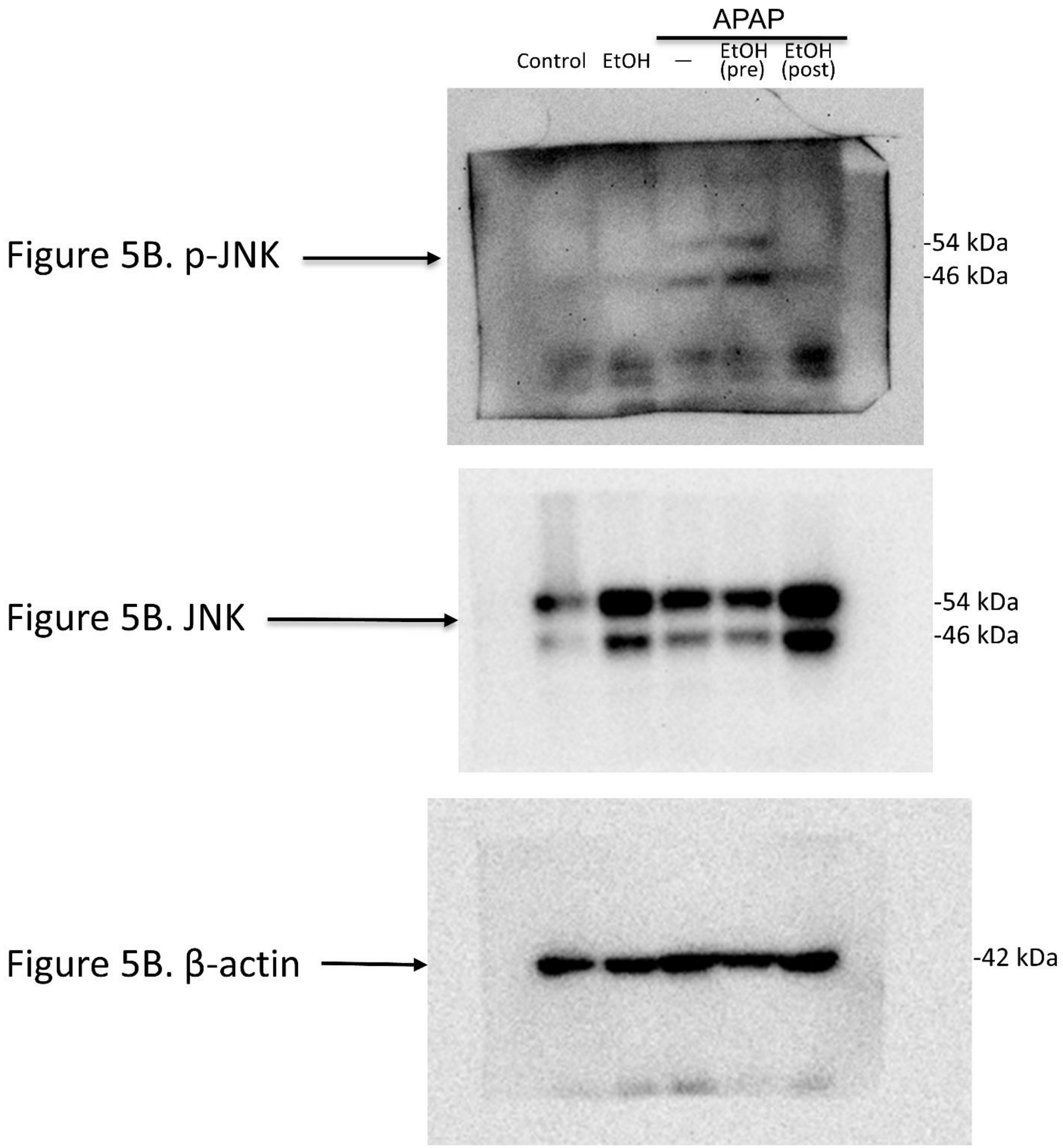

Figure S2. Uncropped Western blots from Figure 5B.

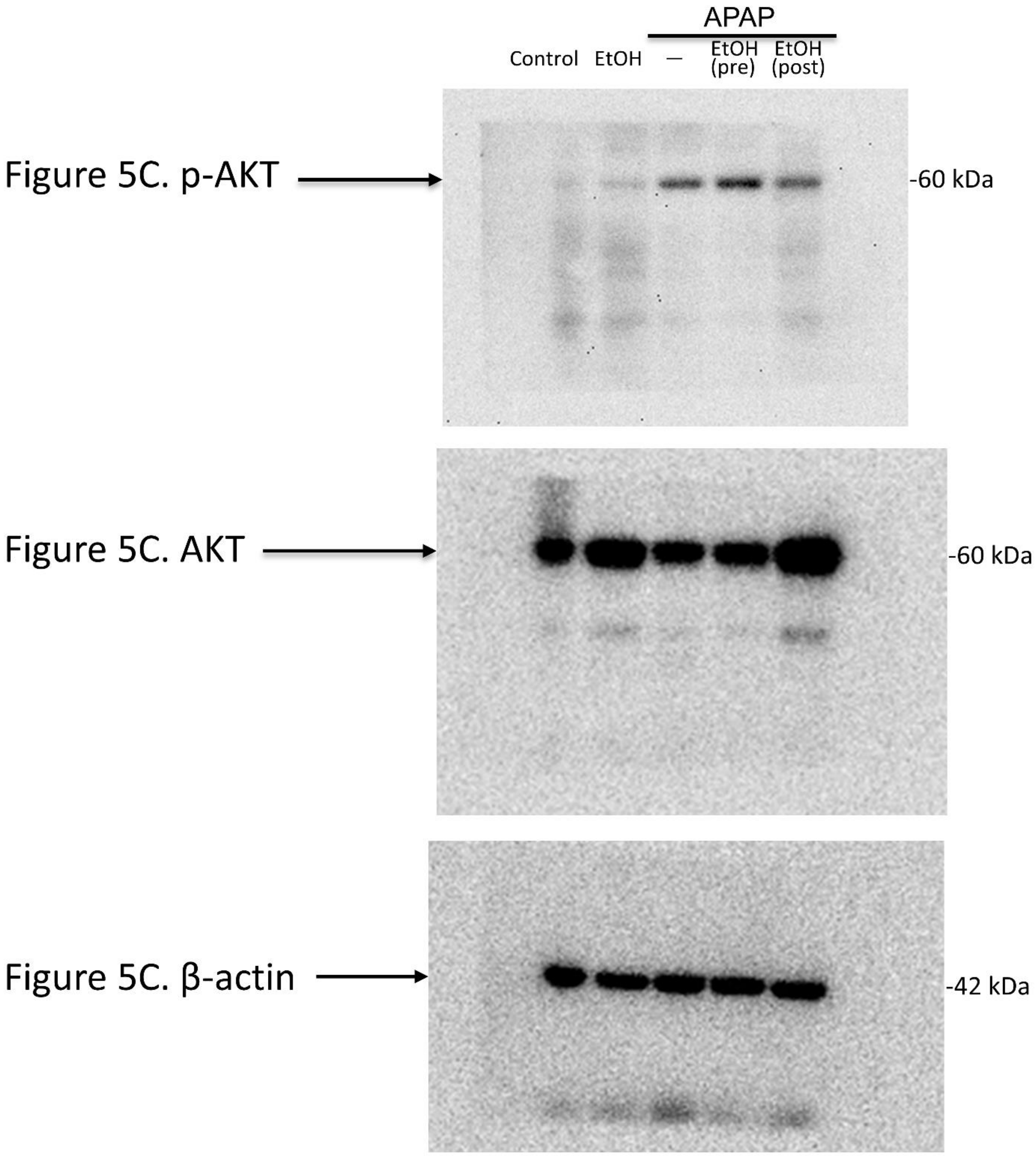

Figure S3. Uncropped Western blots from Figure 5C.

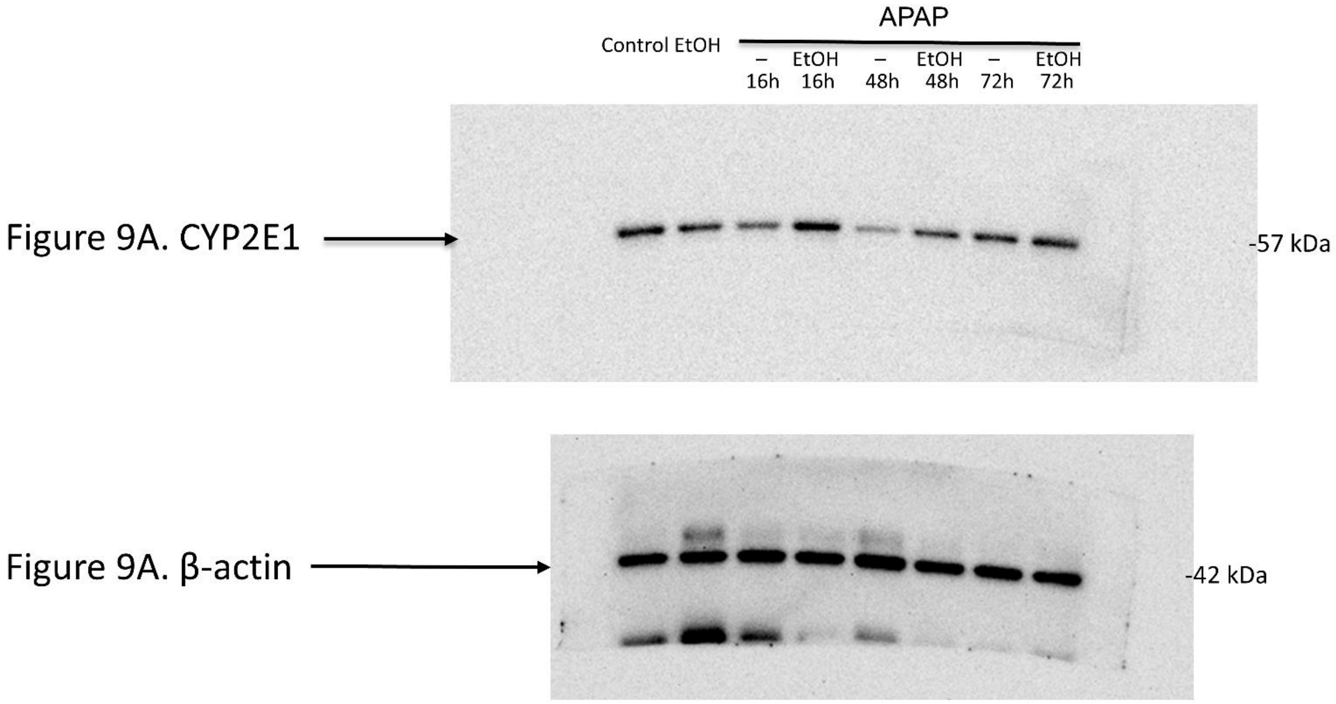

Figure S4. Uncropped Western blots from Figure 9A.

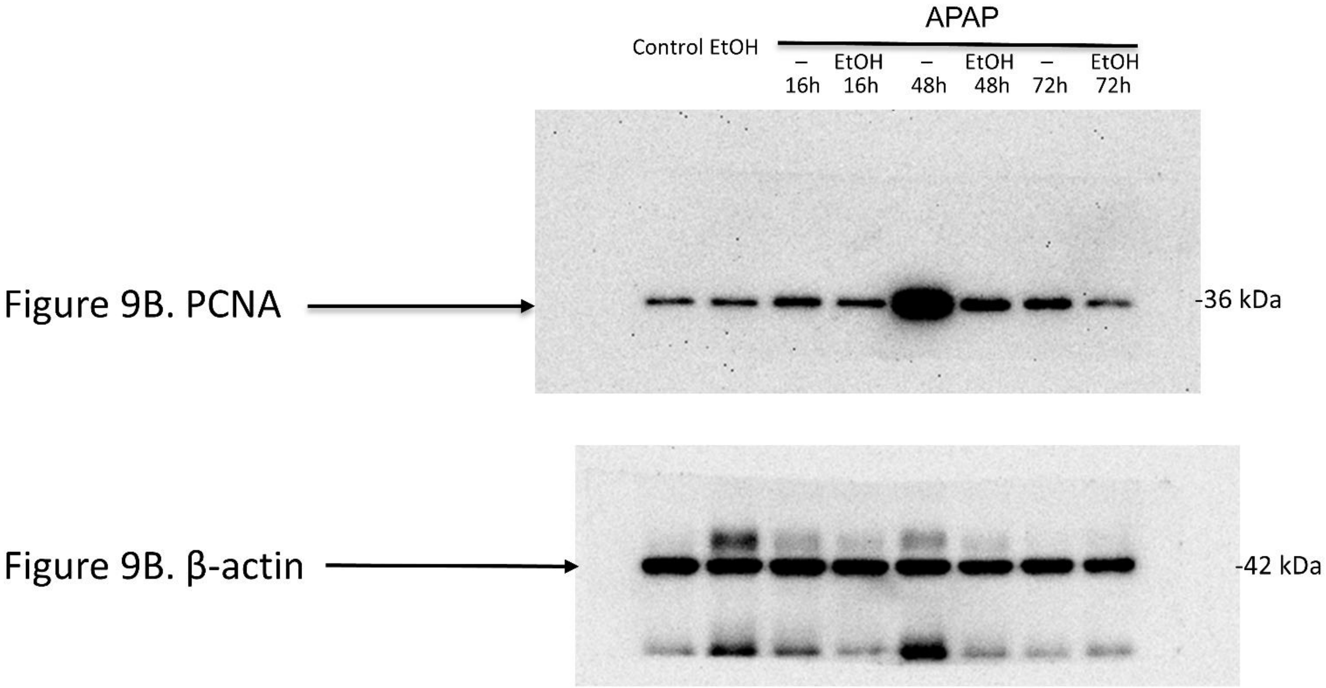

Figure S5. Uncropped Western blots from Figure 9B.
